# Association of Personality Traits and Self-Care Behaviors in People With Type 2 Diabetes Mellitus: A Systematic Review and Meta-Analysis

**DOI:** 10.7759/cureus.50714

**Published:** 2023-12-18

**Authors:** Konstantina Dimou, Elena Dragioti, Georgios Tsitsas, Stefanos Mantzoukas, Mary Gouva

**Affiliations:** 1 Department of Nursing, School of Health Sciences, University of Ioannina, Ioannina, GRC; 2 Department of Economy and Sustainable Development, Harokopio University, Athens, GRC

**Keywords:** type 2 diabetes mellitus, self-management, self-care, personality traits, personality

## Abstract

Diabetes self-care is critical for individuals with type 2 diabetes mellitus (T2DM), and exploring the impact of personality traits on this domain remains pivotal. This study aimed to investigate the association between personality traits and various dimensions of self-care in people with T2DM. A Preferred Reporting Items for Systematic Reviews and Meta Analyses (PRISMA)-guided systematic review with meta-analysis was conducted. Two reviewers independently screened articles, extracted data, and assessed the risk of bias. Estimates were pooled using random-effects meta-analysis. Twenty-three studies, that met our inclusion criteria, revealed distinct associations between certain personality traits and various aspects of self-care. Notably, traits such as openness, conscientiousness, and agreeableness showed associations with improved foot care compliance (odds ratio (OR) = 2.53, 95% CI = 1.49-4.28; OR = 1.84, 95% CI = 1.10-3.08; and OR = 2.07, 95% CI = 1.23-3.48, respectively). Openness was also linked to better overall self-care behaviors (OR = 2.00, 95% CI = 1.17-3.41), while conscientiousness correlated with reduced smoking (OR = 0.96, 95% CI = 0.93-0.99), and agreeableness was associated with improved medication adherence (OR = 1.68, 95% CI = 1.34-2.31). Conversely, traits like extraversion and neuroticism showed associations with decreased medication adherence (OR = 0.77, 95% CI = 0.61-0.96 and OR = 0.51, 95% CI = 0.40-0.65, respectively), with neuroticism additionally linked to lower overall self-care behaviors (OR = 0.67, 95% CI: 0.55-0.81). This study emphasizes the intricate role of personality traits in shaping self-care practices in individuals with T2DM, underscoring the significance of factoring these traits into tailoring and improving diabetes self-care strategies. Nevertheless, establishing definitive causal relationships mandates further in-depth longitudinal investigations and broader meta-analyses to achieve a more conclusive understanding.

## Introduction and background

Burden of type 2 diabetes mellitus

Type 2 diabetes mellitus (T2DM) is a chronic metabolic disease that affects a large proportion of the population, accounting for around 90% of all diabetes cases [1], and is expected to increase [2]. Globally, 415 million people are living with T2DM [3]. Type 2 diabetes mellitus will be the seventh leading cause of death worldwide by 2030 [4], with an estimated 48% of all deaths before the age of 70 [5]. The overall risk of death in people with diabetes is at least double the risk in people without diabetes [6]. T2DM can also cause significant psychological distress [7], common mental health problems such as anxiety, depression, and sleep disorders [8], and can lead to adverse outcomes in people with diabetes [9]. It also impairs cognitive function in several areas, such as attention, concentration, memory, etc., and significantly decreases the quality of life [8]. 

Self-care and personality traits in type 2 diabetes mellitus

The most important therapeutic goal in T2DM is good glycemic control as part of diabetes self-care, which means that people need regular blood glucose monitoring during the day [10,11]. Self-care is an aspect of human life, and it is a complex situation when living with chronic conditions like T2DM. Self-care encompasses the proactive involvement of individuals in overseeing their health and well-being, often in cooperation with healthcare practitioners. It enables individuals to make informed health-related choices, embrace healthy behaviors, and assume accountability for their holistic wellness [12,13]. Within the context of T2DM management, fundamental self-care activities comprise dietary preferences, physical activity, and self-regulation of blood glucose levels, all of which are significantly influenced by cultural and social factors as well as personality traits [14].

Personality traits are enduring and relatively stable patterns of thoughts, emotions, and behaviors that distinguish one individual from another [14]. They represent consistent tendencies in how a person perceives and interacts with the world, responds to situations, and regulates emotions. Personality traits are associated with chronic diseases and can influence health behavior, especially in people with T2DM [12,13], as they play an important role in the patient’s ability to strategize in their lifestyle and self-care [14]. Moreover, personality traits can transform, aggravate, and complicate the disease by predisposing people to convert psychological tensions into physical responses [15]. In other words, personality traits can have both positive and negative implications for T2DM self-care, and some personality profiles, most notably the so-called diabetic personality, which was first proposed in 1963 [16], indicate a poor prognosis, higher risk of medical complications, [8] and general adaptations to T2DM [12].

Hitherto, studies investigating personality traits in individuals with T2DM have primarily concentrated on the five-factor model (FFM) [15], a widely researched framework in personality psychology [14]. The FFM categorizes personality traits into five main dimensions: openness to experience, conscientiousness, extraversion, agreeableness, and neuroticism (also known as the OCEAN model of personality traits). These dimensions offer a layered understanding of an individual, encompassing clusters of traits that often co-occur but are distinct [15]. This model aids in capturing nuanced differences in personality, considered an empirical generalization and a significant concept for personality theorists. Not only is it an empirical generalization, but it is also considered transtheoretical, presenting a crucial phenomenon for personality theorists to elucidate [17]. Aside from the FFM, other influential personality models exist. The Myers-Briggs Type Indicator (MBTI), drawing from Jung's psychological types [18], explores how individuals perceive and engage with the world [19]. Additionally, the HEXACO model introduces six core personality dimensions: honesty-humility, emotionality, extraversion, agreeableness, conscientiousness, and openness to experience [20]. 

Studies have evidenced the FFM's wide applicability across diverse cultures and languages [21,22]. Its influence spans numerous fields, including psychology [23], human factors [24,25], health psychology, school adjustment [26], emotional understanding, evolutionary psychology [27], and career-related aspects such as occupational choice, satisfaction, and performance [25]. Higher levels of conscientiousness, for example, have been associated with potential increases in lifespan by up to five years [28]. Especially concerning the self-care of T2DM, conscientiousness, openness, and neuroticism have been found to be associated with metabolic control of diabetes [29]. Conscientiousness, especially when it comes to physical activity, makes a significant contribution to self-care behavior [30]. It is also confirmed that conscientiousness contributes to better T2DM medication adherence [31]. According to the literature, the way of life may be influenced by personality traits, higher levels of extraversion have been linked to aspects of a diabetes-prone lifestyle, such as unhealthy eating habits and low levels of physical activity [32,33]. Other personality traits have also been associated with self-care dimensions in patients with chronic disease [34,35]. In people with diabetes, type D personality has a negative influence on healthcare visits [36]. However, many other studies have reported conflicting results [37-40].

The present study

Against this background, it is crucial to examine the personality traits of people with T2DM and their role in self-care behavior. Many organizations, such as the Scottish Intercollegiate Guidelines Network and the Institute for Clinical Systems Improvement, have highlighted the importance of personality assessment and psychological interventions in T2DM [41,42]. Even though many systematic reviews have been published to date, they either focus on mental health (e.g., depression or anxiety) or individually on personality traits within mixed diabetes populations (e.g., T1DM, T2DM, or gestational diabetes) or focusing on type 2 diabetes risk [43,44]. A comprehensive portrait of the role of personality traits in self-care behavior by meta-analytic methods is lacking. Therefore, the authors conducted this systematic review with meta-analysis to examine whether certain personality traits inside and outside the FFM are associated with self-care behaviors and management in people with T2DM.

## Review

Methods

Study Design

The authors followed the Preferred Reporting Items for Systematic Reviews and Meta-Analysis checklist (PRISMA) [45] and the guidelines for Meta-Analyses and Systematic Reviews of Observational Studies (MOOSE) [46]. The protocol for this review was prospectively registered in the International Prospective Register of Systematic Review (PROSPERO; CRD 42021291229). This article differs from the full protocol in that it focuses solely on personality traits as part of a larger systematic review.

Search Strategy

We searched PubMed/Medline and APA for published articles in peer-reviewed journals from inception through January 9, 2023. The basic search strategy included a combination of keywords or terms related to personality traits or personality characteristics, type 2 diabetes mellitus, self-care, and self-management using the Boolean operators “AND” and “OR” to combine them. The full search query for all databases is reported in Appendix Table 1.

No restrictions regarding the year of publication, country, ethnicity, or any other characteristic applied during the search process. Additionally, reference lists of previously published work were screened to identify other potentially relevant articles.

One author screened the titles and keywords of each article for eligibility. Those who met the initial screening criteria were then screened at the abstract level. If a study appeared to meet our eligibility criteria, the full text was obtained and reviewed by two independent authors. Discrepancies were discussed and resolved through discussions by the research team.

Eligibility Criteria

The authors included peer-reviewed articles examining the association between personality traits or personality traits and self-care in adults (aged 18 and older) with T2DM using a cohort, cross-sectional, and case-control study design, randomized control trials (RCTs) were also included when personality was reported at baseline. The authors mainly focused on the Big Five-factor model (i.e., openness, conscientiousness, extraversion, agreeableness, neuroticism; OCEAN model) [47]. However, the authors expanded the definition to include other common personality traits such as type A and type D personality, optimism, self-esteem, narcissism, and honesty-humility, etc. [48]. Eligible articles included people with T2DM diagnosed by a physician according to the American Diabetes Association (ADA) and International Diabetes Federation (IDF) criteria [1,49]. These criteria include ≥6.5 A1C random plasma glucose ≥200mg/dL (11,1 mmol/L), and hyperglycemia. Articles reporting solely on people diagnosed with major complications (retinopathy, diabetic foot, coronary heart disease, etc.) or on people with a clinical psychiatric diagnosis were deemed ineligible. Because the authors were interested in self-care behavior, they focused on the following outcomes: self-care and management.

Articles were excluded if they: 1) involved people with type 1 diabetes mellitus (T1DM) or mixed populations as noted above, 2) did not report how T2DM was diagnosed or how personality traits/characteristics were assessed 3) reported on people with serious complications of T2DM 4) were systematic reviews, intervention studies, pilot studies, qualitative studies, and mixed-method studies, 5) reported associations other than personality traits, e.g., depression, anxiety etc., or biomarkers or inflammatory mechanisms, and 6) reported on other outcomes as described above. In the case where the article includes data on people with mixed diabetes conditions, we only considered inclusion if separate results were reported for T2DM. Studies on people with prediabetes and studies published in languages other than English were excluded.

Quality Assessment

The authors assessed the methodological quality of the included studies using the Newcastle-Ottawa Scale (NOS) tool for observational studies. The NOS tool can be used for cohort, case-control, and cross-sectional studies. It consists of a set of criteria that must be considered by assigning a star scoring system from 0-10 to the following tree domains: 1) Selection 2) Comparability and 3) Outcome per study design [50]. In the case of RCTs, the authors used the revised Cochrane Risk of Bias (RoB 2.0) tool [51]. The quality assessment was evaluated by one author (KD) and checked by the other two team members (ED and MG). Disagreements were discussed and resolved through discussions within the research team.

Data Extraction

All citations obtained using the search strategy were imported into Endnote, and duplicates were removed. The authors extracted data into a pre-defined Excel spreadsheet. The list of variables of interest included PubMed identifier/digital object identifier (PMID/DOI), first author, year of publication, country, design of included studies (cohort, cross-sectional, etc.), sample size, diabetes medication (if reported), mean age, duration of diabetes, diabetes diagnostic criteria, % of women, personality traits and their measurement tools (e.g., the Revised NEO Personality Inventory (NEO PI-R) etc.), outcomes and their measurement tools (e.g., Diabetes Self-Care Scale (C-DSC) etc.), the main findings of each study. The authors also recorded any quantitative data on the association between personality traits and self-care activities (e.g., odd ratios (ORs), with their 95% confidence intervals (CIs), correlation coefficients, or any other indirect information needed to estimate the association measure).

Data Synthesis

The results were structured according to personality traits in relation a) to the Big Five- factor model (i.e., OCEAN model of personality traits) and b) other personality traits (i.e., traits outside of the five-factor model, e.g., optimism, impulsivity, type D personality, etc.) to comprehend the relationship between personality and self-care behaviors. For studies reporting appropriate statistics, a meta-analysis using a random effects model, due to expected high heterogeneity between studies, [52] with the Hartung-Knapp-Sidik-Jonkman method was performed [53]. This method outperforms when the number of studies is small [54]. There was methodological heterogeneity, with studies using different measures of associations. To harmonize the data for the meta-analysis, we converted the regression and/or correlation coefficients to standardized mean differences [55] and then converted them to log-odds ratios (logORs) and standard errors (SElogORs) [56]. For binary outcomes, ORs and SEs were transformed into logORs and SElogORs. Finally, the authors pooled logORs and SElogORs of each study to produce summary effect sizes in a forest plot as an OR with 95% confidence intervals (CI) [57]. Heterogeneity of findings was assessed using χ2, t2, and I2 statistics [52]. Publication bias was assessed using the Eggers test (p-value < 0.10) [58] only when any outcome was reported in 10 or more included meta-analyses [59]. Data analysis was conducted using Stata V.17.0 and all analyses with p < 0.05 were interpreted as significant.

Results

**Figure 1:**
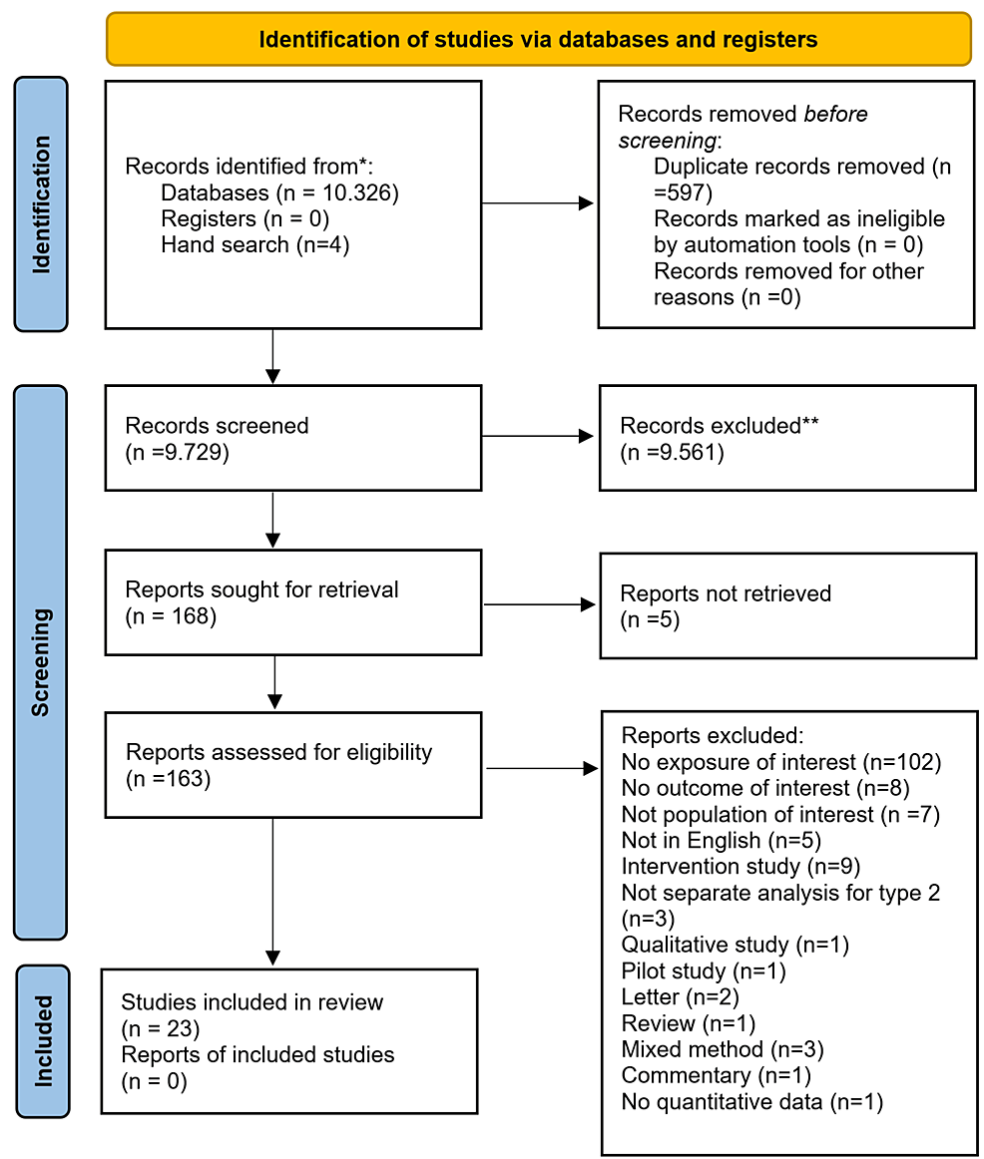
Study selection flowchart

Initially, the authors identified 10,326 records from the two databases and four studies by hand search. After deduplication (n=597), a total of 9,729 records were examined for eligibility. The authors further excluded 9,561 articles based on title and abstract, leaving a total of 168 studies that were read in full text, except for five articles whose full text could not be retrieved. One hundred and sixty-three studies were scrutinized for depth eligibility, and finally, 23 primary studies, involving a total of 8378 people with T2DM, met our inclusion criteria and were included in this systematic review and meta-analysis [7,11-13,15,29-31,36,37,39,40,60-70]. The selection process is presented in Figure 1.

Characteristics of included studies

The characteristics of the included studies are summarized in Appendix Table 2. All the included studies were published from 1984 to 2022. Seven studies were conducted in the United States [29,31,40,61,65,68,69], 11 in Asia [7,13,15,37,39,62-64,66,67,70], one in Mexico [12], two in Europe [36,60], and two in Australia [11,30]. A total of 15 studies (66%) were cross-sectional, six (26%) were longitudinal cohorts, while one (4%) was RCT, and one (4%) had a within-subjects repeated measurement study design.

The median number of participants per study was 199, with a (interquartile range (IQR)) of 95-361. The median mean age of participants was 58 years (IQR=53-62) and the median percent of females was 54 (IQR=43-65). The duration of T2DM ranged between under six months to 14 years and most studies (n=17) included people with a known diagnosis of T2DM, while only four studies used either ICD-10 or ADA (2016) criteria. The medications utilized encompassed oral antidiabetic medications, insulin injections, or a combination of both treatments. However, the provided information was not thoroughly documented. Approximately half of the studies included in the analysis provided data on medications [7,11,15,31,37,39,40,61,63-65,70], whereas 11 studies lacked information on medication specifics [12,13,29,30,36,60,62,66-69].

Of 23 studies, 13 (57%) studies assessed personality traits within the Big Five Factor model, including tools such as the Big Five Inventory (BFI), the Five Factor Personality Inventory (FFPI) or the Revised NEO Personality Inventory (NEO PI- R) [11,12,29-31,37,40,63,65,66,68,70]. For personality traits outside of the Big Five model, five (22%) studies assessed Type D personality using the Type D scale (DS-14) [7,13,36,39,67], two (8%) assessed self-esteem using the Rosenberg Self-Esteem Scale (RSES) [60,64] and one study (4%) each examined Type A personality, impulsiveness, narcissism, optimism, angry hostility, vulnerability, and altruism [60-62,65,69]. In the context of diabetes self-care, 16 studies focused on glycemic control by using glycated hemoglobin (A1C), seven on physical activity, six on medication adherence, five on diet, two on foot care, and one on overall adherence to self-care activities by mainly using the Summary of Diabetes Self-Care Activities measure (SDSCA) or the Diabetes Self-Care Scale (C-DSC).

Summary of studies' quality

The results of the included studies’ quality assessment are presented in Appendix Table 2. More than half of the studies (74%; n=17) had good/moderate quality, while only six (26%) had high quality.

Associations between five-factor-model (or OCEAN) personality traits and self-care activities

Openness

**Figure 2:**
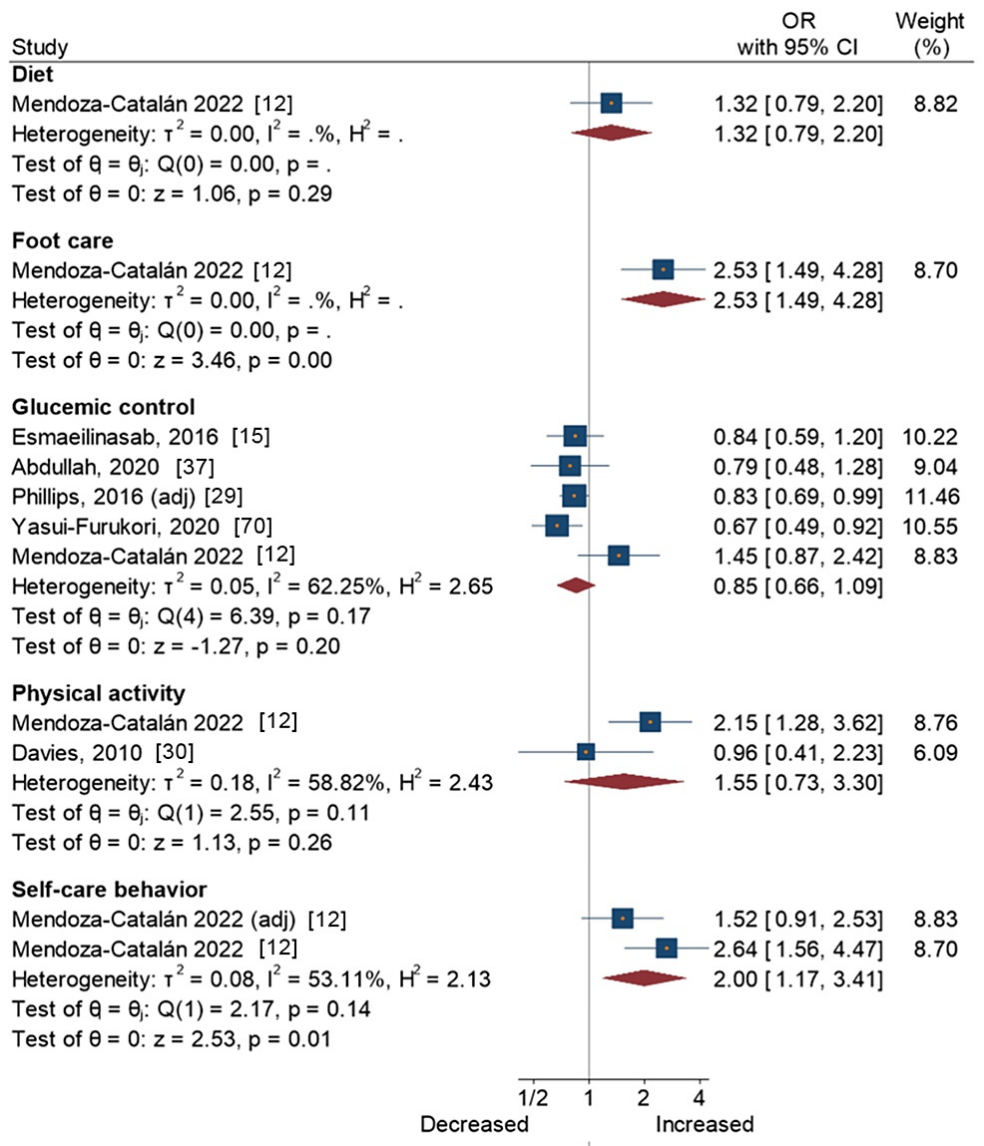
Openness and self-care activities in people with type 2 diabetes Mendoza-Catalán, 2022 [12]; Esmaeilinasab, 2016 [15]; Abdullah, 2020 [37]; Phillips, 2016 [29]; Yasui-Furukori, 2020 [70]; Davies, 2010 [30]

Figure 2 presents a forest plot with effect sizes and 95% CIs for the associations between openness and self-care activities. Results from one study [12] showed that openness was associated with greater compliance with foot care (OR = 2.53, 95% CI = 1.49-4.28) and overall self-care behavior (OR = 2.00, 95% CI = 1.17-3.41). Meta-analytic pooling of five studies on the associations between openness and glycemic control yielded a non-significant summary OR of 0.85 (95% CI, 0.66-1.09), with large heterogeneity across the studies (I2 = 62%; τ2 = 0.05; P = 0.17). This was also the case for the associations between openness and other self-care activities, i.e., diet and physical activity (Figure 2).

Conscientiousness

**Figure 3:**
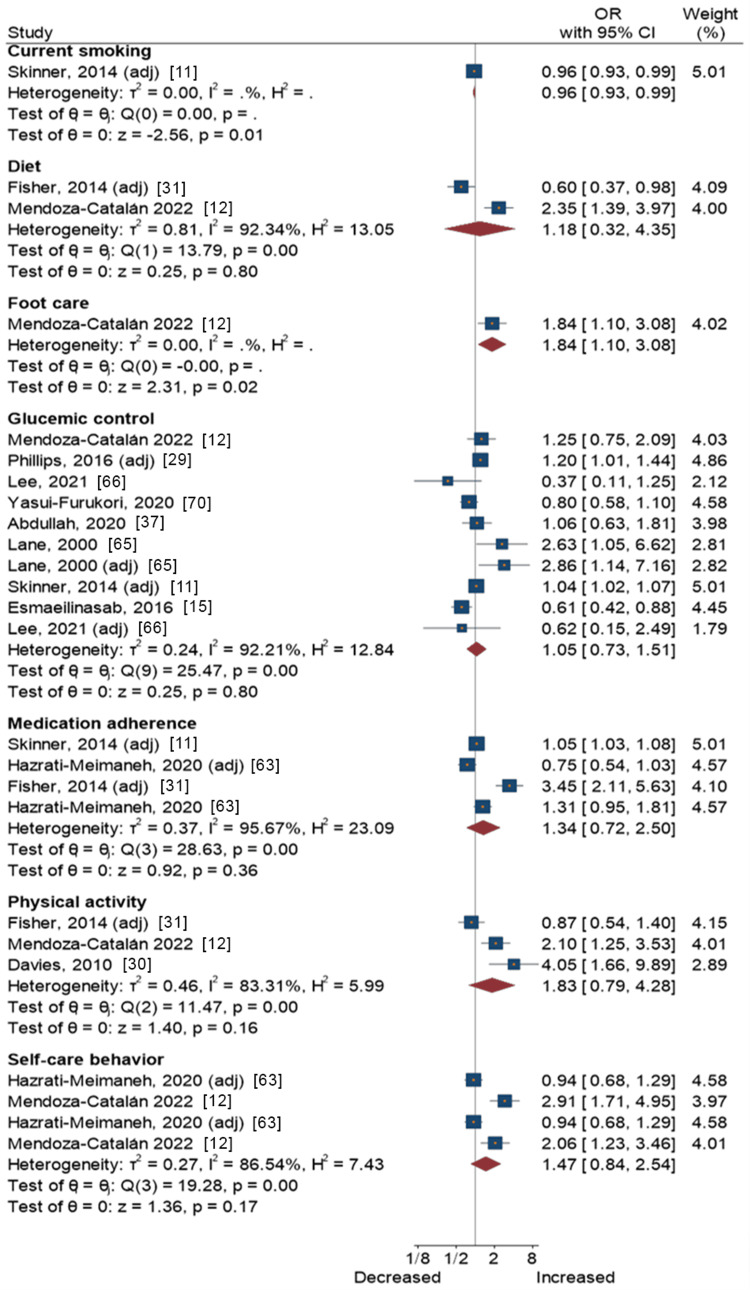
Conscientiousness and self-care activities in people with type 2 diabetes Skinner et al., 2014 [11]; Fisher et al., 2014 [31]; Mendoza-Catalán et al., 2022 [12]; Phillips and Guarnaccia, 2016 [29]; Lee and Li, 2021 [66]; Yasui-Furukori et al., 2020 [70]; Abdullah et al., 2020 [37]; Lane et al., 2000 [65]; Esmaeilinasab et al., 2016 [15]; Hazrati-Meimaneh et al., 2020 [63], Davies et al., 2010 [30]

Figure 3 presents a forest plot with effect sizes and 95% CIs for the associations between conscientiousness and self-care activities. Results from one study [11] showed that conscientiousness was associated with lower odds of current smoking (OR = 0.96, 95% CI = 0.93-0.99). Another study found [12] that conscientiousness was associated with greater compliance with foot care (OR = 1.84, 95% CI = 1.10-3.08). Meta-analytic pooling of the associations between conscientiousness and other self-care activities, i.e., diet, glycemic control, medication adherence, physical activity, and overall self-care behavior, yielded non-significant associations (Figure 3).

Extraversion

**Figure 4:**
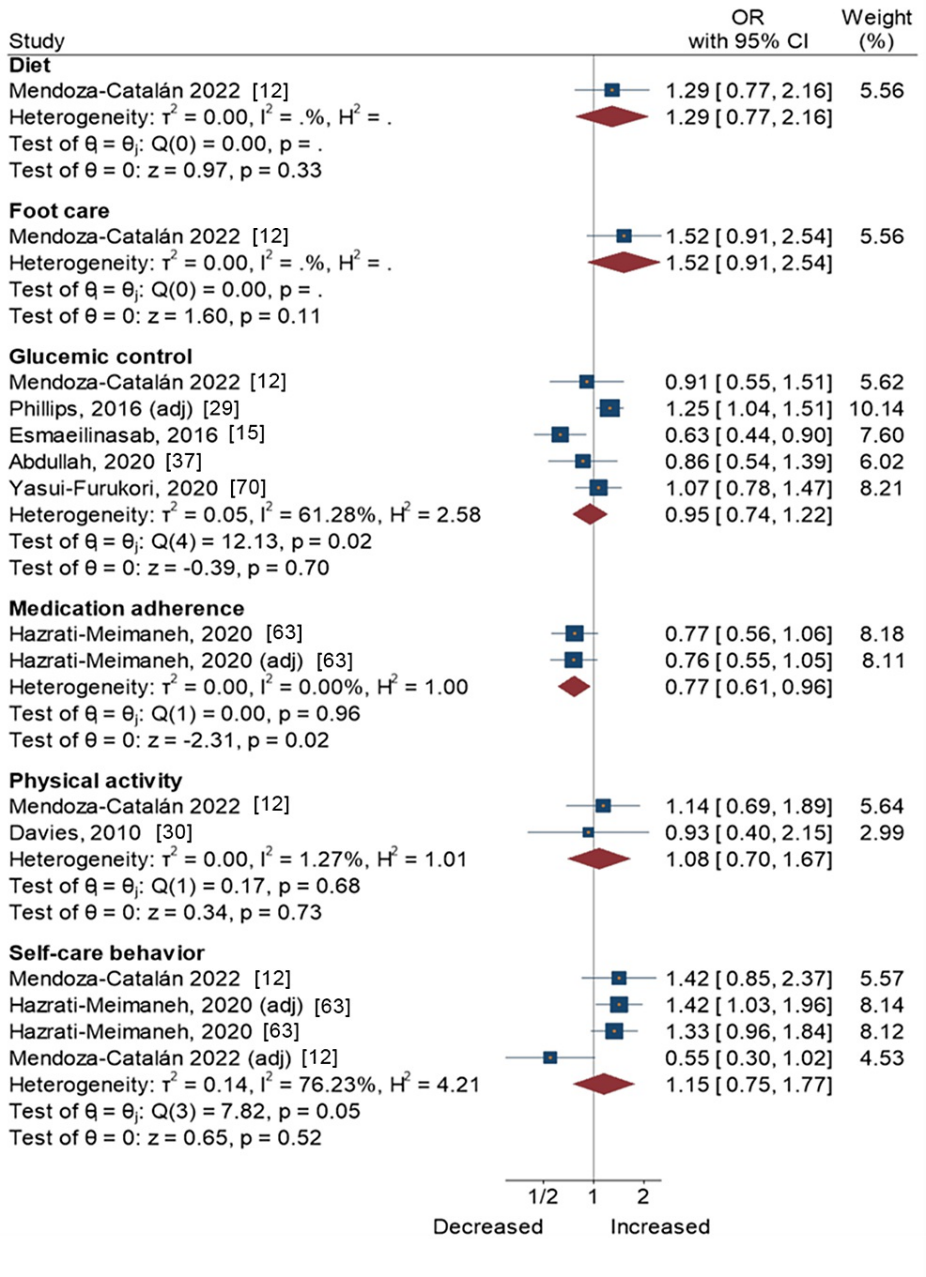
Extraversion and self-care activities in people with type 2 diabetes Mendoza-Catalán et al., 2022 [12]; Phillips and Guarnaccia, 2016 [29]; Esmaeilinasab et al., 2016 [15]; Abdullah et al., 2020 [37]; Yasui-Furukori et al., 2020 [70]; Hazrati-Meimaneh et al., 2020 [63]; Davies et al., 2010 [30]

Figure 4 presents a forest plot with effect sizes and 95% CIs for the associations between extraversion and self-care activities. Results from one study [63] showed that extraversion was associated with lower medication adherence (pooled OR = 0.77, 95% CI = 0.61-0.96). However, this was not the case for extraversion, diet, and foot care [12]. Meta-analytic pooling of the associations between extraversion and other self-care activities, i.e., glycemic control, physical activity, and overall self-care behavior, also yielded non-significant associations (Figure 4).

Agreeableness

**Figure 5:**
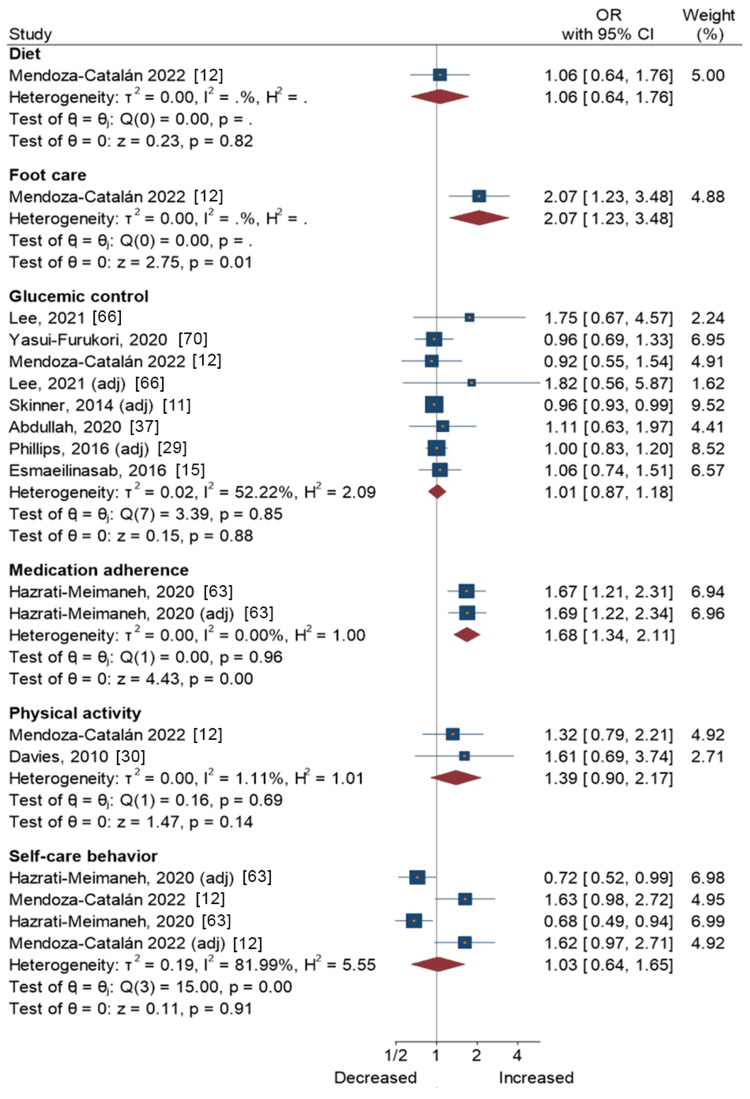
Agreeableness and self-care activities in people with type 2 diabetes Mendoza-Catalán et al., 2022 [12]; Lee and Li, 2021 [66]; Yasui-Furukori et al., 2020 [70]; Skinner et al., 2014 [11]; Abdullah et al., 2020 [37]; Phillips and Guarnaccia, 2016 [29]; Esmaeilinasab et al., 2016 [15]; Hazrati-Meimaneh et al., 2020 [63]; Davies et al., 2010 [30]

Figure 5 presents a forest plot with effect sizes and 95% CIs for the associations between agreeableness and self-care activities. Results from one study [12] showed that agreeableness was associated with greater compliance with foot care (OR = 2.07, 95% CI = 1.23-3.48), whereas no association was found between agreeableness and diet. Another study found that agreeableness was associated with greater medication adherence (OR = 1.68, 95% CI = 1.34-2.31) [63]. Meta-analytic pooling of the associations between agreeableness and other self-care activities, i.e., glycemic control, physical activity, and overall self-care behavior, yielded non-significant associations (Figure 5).

Neuroticism

**Figure 6:**
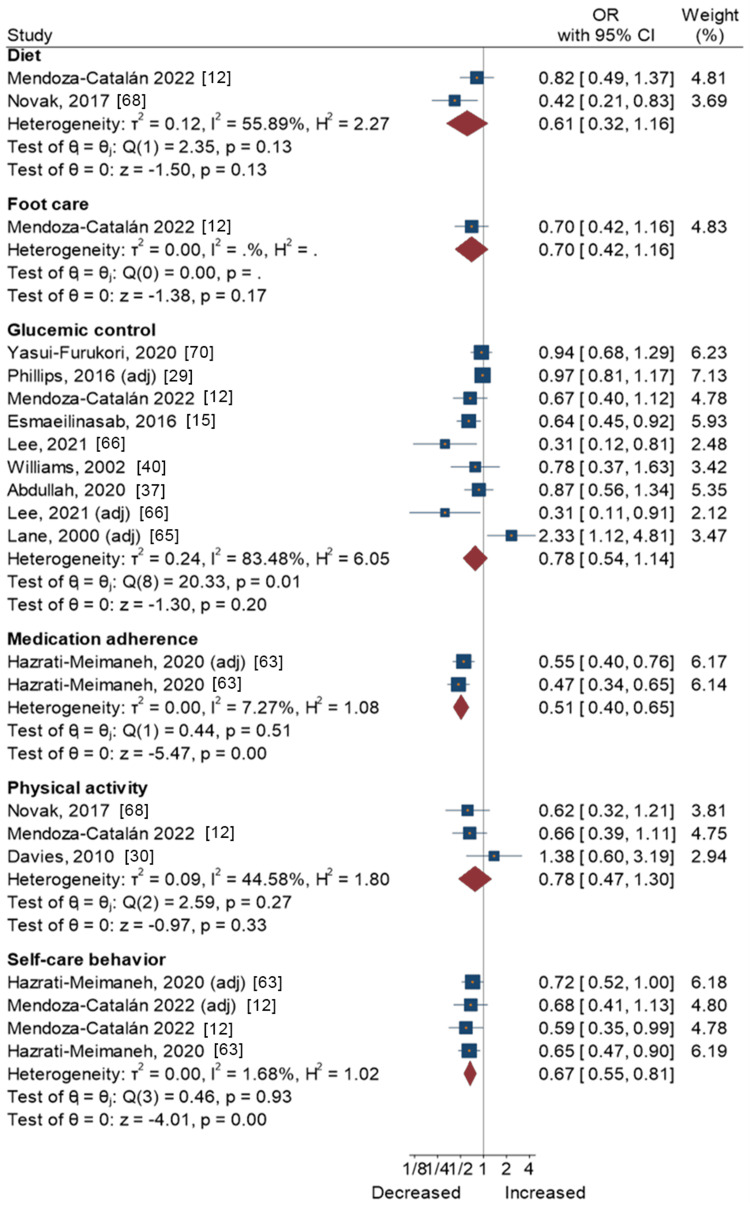
Neuroticism and self-care activities in people with type 2 diabetes Mendoza-Catalán et al., 2022 [12]; Novak et al., 2017 [68]; Yasui-Furukori et al., 2020 [70]; Phillips and Guarnaccia, 2016 [29]; Esmaeilinasab et al., 2016 [15]; Lee and Li, 2021 [66]; Williams et al., 2002 [40]; Abdullah et al., 2020 [37]; Lane et al., 2000 [65]; Hazrati-Meimaneh et al., 2020 [63]; Davies et al., 2010 [30]

Figure 6 presents a forest plot with effect sizes and 95% CIs for the associations between neuroticism and self-care activities. Results from one study found that neuroticism was associated with lower medication adherence (OR = 0.51, 95% CI = 0.40-0.65) [63]. Meta-analytic pooling of two studies on the associations between neuroticism and overall self-care behavior yielded a significant summary OR of 0.67 (95% CI, 0.55-0.81), with low heterogeneity across the studies (I2 = 1.68%; τ2 = 0.00; P = 0.93), indicating lower compliance with self-care among those with high neuroticism. However, this was not the case for neuroticism and other self-care activities, i.e., diet, foot care, glycemic control, and physical activity (Figure 6).

Associations between other personality traits and self-care activities

Appendix Figures 7-13 present forest plots with effect sizes and 95% CIs for the associations between other personality traits and self-care activities. Results from four different studies [7,13,36,39] found that type D personality was associated with lower diabetes management self-efficacy (OR = 0.22, 95% CI = 0.15-0.33), compliance with diet (OR = 0.36, 95% CI = 0.21-0.61), foot care (OR = 0.37, 95% CI = 0.22-0.63), medication adherence (OR = 0.28, 95% CI = 0.19-0.42), physical activity (OR = 0.53, 95% CI = 0.32-0.88), overall self-care behavior (OR = 0.33, 95% CI = 0.19-0.57), and lower frequency of visits to the primary care physician (OR = 0.37, 95% CI = 0.15-0.94). Meta-analytic pooling of the association between type D personality and glycemic control yielded a non-significant association (Appendix Figure 7).

Results from one study [65] found that altruism was associated with poorer glycemic control (OR = 0.22, 95% CI = 0.09-0.57), while angry hostility was associated with better glycemic control (OR = 2.38, 95% CI = 1.14-4.97). Meta-analytic pooling of two studies on the associations between self-esteem and glycemic control yielded a significant summary OR of 1.77 (95% CI, 1.16-2.70), with low heterogeneity across the studies (I2 = 0.02%; τ2 = 0.00; P = 0.88), indicating a better glycemic control among those with high self-esteem (Appendix Figure 8).

Further results from one study [69] found that impulsivity was associated with lower compliance with diet (OR = 0.29, 95% CI = 0.10-0.85) [69]. No other significant associations were found (Appendix Figures 9-13).

Subgroup analysis and assessment of publication bias

Due to an insufficient amount of data, neither a subgroup analysis for characteristics at the study level (e.g., sex of the participants, adjusted vs unadjusted estimates, etc.) nor an assessment of the publication bias could be carried out.

Discussion

Main Findings

In this systematic review with meta-analysis, the authors explored the intricate relationship between personality traits, encompassing both those within and outside the five-factor model [17,71], within the context of T2DM, specifically focusing on their influence on self-care behaviors. Self-care in chronic conditions, particularly T2DM, poses a multifaceted challenge requiring continuous management [72]. Managing diabetes entails vigilant monitoring of blood glucose levels, a critical component for achieving optimal glycemic control, alongside necessary adjustments in lifestyle such as diet and physical activity [10,11]. In this study, intriguing insights emerged: personality traits like openness, conscientiousness, and agreeableness exhibited potential positive associations with various facets of self-care, including effective foot care management, overall self-care practices (including medication adherence), and a reduced propensity for smoking among individuals with T2DM. Conversely, traits such as extraversion and neuroticism showcased adverse effects on medication adherence and overall self-care behaviors. However, aside from a few isolated instances, our outcomes did not definitively establish an autonomous correlation between personality traits and enhanced glycemic control. Particularly, the impact of type D personality appears more conspicuous across several dimensions of self-care behavior. Additionally, considerable diversity was observed among studies regarding their objectives, methodologies, and measurement approaches. Overall, this study underscores the influence of personality traits on health-related behaviors, significantly impacting an individual's ability to manage their well-being through lifestyle choices and self-care practices. Past studies have consistently highlighted the role of personality in shaping health behaviors and influencing the management of health status through lifestyle and self-care [17,71,73]. The health-related behaviors and personal health choices of individuals with T2DM are also influenced by their personality traits [73,74].

Personality Trait Findings in the Context of the Five-Factor Model for T2DM Self-Care

Openness is partly related to self-care in T2DM [12] and specifically linked to high compliance with foot care and general self-care behaviors, but it yielded non-significant associations with glycemic control, diet, medication adherence, and physical activity. The inconsistent associations between openness and objective markers of glycemia or glycemic control are supported by previous work [29,75] but not for physical activity compliance [76]. This finding is very interesting as openness is a personality factor that is particularly related to adaptive coping [77] by reducing many of the negative short-term physical effects of environmental stressors. People with openness are described as curious, as people with open feelings are open to new and different ideas, and openness to experience refers to proneness to novelty [78]. It could be argued that while being diagnosed with a chronic disease such as diabetes may be a new experience and a new way of life for those with greater openness, [79] it should be noted that there can be a sliding scale of openness as such some people are with T2DM more receptive to certain self-care activities than others.

Compared to previous individual studies [29,31], the authors found that conscientiousness was only associated with greater foot care compliance [12,30]. Of particular note is the positive association between conscientiousness and the ability to quit current smoking [11]. It has been suggested that conscientiousness is a dutiful, achievement-oriented, orderly, and accepting of the rules trait [80], and therefore, people having this trait may show greater compliance with self-care activities and risk of diabetes [75,81,82]. Many other individual studies of people with chronic conditions other than diabetes consistently also found positive health benefits of being highly conscientious [76,83-85]. The observed results are also consistent with the conclusions of a systematic review that included 17 studies of adults with type 2 diabetes and found weak to moderate associations between weight management and personality traits such as neuroticism and conscientiousness [73]. However, our meta-analytic findings on associations between conscientiousness and self-care behaviors, including glycemic control, did not confirm any significant associations; a finding worth investigating further.

This also applied to the associations with extraversion, agreeableness, and neuroticism. Results from individual studies showed that agreeableness was positively associated with foot care compliance [12] and medication adherence [63], while extraversion and neuroticism were the opposite [63]. However, neuroticism also had a negative association with overall self-care behavior, after the meta-analytic pooling of two studies [12,63]. These results are consistent with the literature, and several pathways may explain these associations. The agreeableness trait includes people who are trusting, compliant, modest, and straightforward [86] and are therefore more likely to adhere to medication for chronic conditions by maintaining a constructive attitude [87]. Extraversion is closely linked to a diabetes-prone lifestyle, urging the patient to adopt unhealthy eating habits and a more sedentary lifestyle accompanied by the absence of physical activity [32,33]. Neuroticism is a personality trait that makes people feel high-stress reactivity and experience negative emotions [88], which is considered a risk factor for depression in people with T2DM, and makes them feel less emotionally controlled [89], which negatively influences self-care compliance. Moreover, neuroticism is associated with poorer patient outcomes and contributes to anxiety and anhedonia in people with chronic illnesses such as major depressive disorder [90]. However, non-significant associations were found from the meta-analytic summary of associations between neuroticism and self-care behaviors. This also prompts further investigation, as higher levels of neuroticism are consistently associated with poor health outcomes [91].

Personality Trait Findings T2DM Self-Care Beyond the Five-Factor Model Context

Apart from the FFM personality traits, our study revealed that individuals with a type D personality exhibited shortcomings in managing their diabetes. Specifically, they displayed poor adherence to various self-care behaviors such as diet, foot care, and medication compliance, along with reduced engagement in physical activity, and fewer visits to their primary care physician [7,13,36,39]. These findings align with existing literature, indicating that individuals grappling with chronic illnesses alongside heightened negative emotions and social inhibitions encounter challenges in managing their health effectively. This group tends to struggle in monitoring their health status and adhering to healthy practices, including maintaining regular physical activity or following a balanced diet [10,92,93]. It's essential to explore further whether a stress-related personality, previously noted in cardiovascular diseases, also manifests in individuals with T2DM. Notably, our meta-analysis found no evidence supporting a link between type D personality and poor glycemic control. Additionally, impulsivity was associated with reduced adherence to dietary plans. While it's unsurprising that high self-esteem correlates with better glycemic control [60,64], it's intriguing that angry hostility displayed a similar association, indicating improved glycemic control. Conversely, altruism was linked to poorer glycemic control [65]. For the latter, it has been suggested [65] that people high in altruism tend to focus on the needs of others rather than themselves and are therefore at increased risk of lack of self-care, while those with negative emotions might tend to focus on their needs, which increases their motivation to achieve better self-care control.

Strengths and limitations

Our study stands as one of the few systematic reviews utilizing meta-analysis to investigate personality traits and self-care behaviors in individuals with T2DM, which enhances the accuracy of the findings presented. Previous systematic reviews have mainly leaned towards narrative synthesis or explored outcomes other than self-care practices [73,74]. This is of great importance considering the growing importance of self-care for people with chronic health conditions in modern healthcare systems [87]. The fact that none of our included studies were of low quality is an additional strength.

However, this study has some notable limitations. First, it should be noted that our significant findings are predominantly based on a small number of individual studies, usually one or two, for each aspect of self-care. In most cases, the combined summary estimates lacked substantial evidence to draw significant conclusions (e.g., summary OR for the association between openness and glycemic control). The primary reason for this stems from the scarcity of individual studies available for pooling across discrete self-care activities. Furthermore, the association did not indicate a causal relationship, even considering that the majority of included studies had a cross-sectional study design. Taken together, our findings may suggest that individual studies are likely to overestimate the associations between personality traits and better self-care behaviors, compared to aggregated estimates.

In this regard, future well-conducted longitudinal studies and more subsequent meta-analyses are recommended to confirm the above hypothesis. Converting the various metrics to logORs had the advantage of allowing direct comparison between studies, but resulted in larger confidence intervals and thus, there could be an error in the estimates.

Another limitation pertains to potential language bias, as our review exclusively incorporated studies published in English owing to constraints in accessing precise and comprehensive translation resources. Additionally, our focus was solely on T2DM, neglecting other diabetes types such as Type 1 Diabetes or gestational diabetes. Moreover, our study concentrated specifically on personality traits and did not encompass other psychological variables such as anxiety or depression. Furthermore, due to inadequate reporting within the studies included in our analysis and the relatively limited number of comparisons available, we encountered challenges in exploring additional factors that might have influenced our findings (e.g., publication bias, type of diabetic medication, or age and sex differences, etc.).

Future research

Moving forward, several prospective areas merit exploration to comprehensively understand the interplay between personality traits and self-care behaviors in individuals with T2DM. A crucial step forward involves conducting more robust longitudinal studies with larger cohorts to explore associations between personality traits and various dimensions of self-care in T2DM. This approach would enable a comprehensive understanding of how these traits evolve and how they dynamically impact long-term self-care practices. A higher quantity of well-designed studies can also strengthen the reliability of conclusions drawn from meta-analyses, reducing the potential overestimation seen in individual study analyses. Moreover, further meta-analyses are warranted to consolidate evidence by pooling data from diverse longitudinal studies, facilitating more accurate estimations and reducing potential biases inherent in smaller, individual studies. To mitigate language bias, future research should aim to include studies in languages other than English, providing a more global perspective. Moreover, while focusing primarily on T2DM provided clarity in our study, encompassing other diabetes types, such as Type 1 Diabetes and gestational diabetes, might provide a broader context and comparison. Furthermore, broadening the scope to encompass a wider array of psychological variables like anxiety, depression, and stress could provide a more comprehensive understanding of their influence on self-care behaviors. Improvements in reporting standards within studies are vital. Encouraging more detailed and consistent reporting of methodologies, including demographics, types of medications, and other influential factors, would significantly enhance future meta-analytic studies. Furthermore, exploring additional factors like publication bias, variations in types of diabetic medications, and demographic disparities such as age and gender could provide valuable insights into the nuanced relationships between personality traits and self-care behaviors. These future directions hold promise in advancing our comprehension and refining interventions for better management of T2DM through tailored and personalized approaches.

## Conclusions

Our systematic review, incorporating both the FFM personality traits and other dimensions, sheds light on varied associations between personality traits and self-care activities in this context. The findings unveiled intriguing associations between personality traits and different aspects of self-care. Notably, traits like openness, conscientiousness, and agreeableness showed potential positive links with specific self-care dimensions, such as effective foot care management, overall self-care practices (including medication adherence), and reduced smoking tendencies. However, these traits didn't significantly correlate with enhanced glycemic control, diet, or physical activity. In contrast, traits like extraversion and neuroticism exhibited adverse effects on certain self-care behaviors, particularly medication adherence and overall self-care practices. While neuroticism showed lower compliance across self-care behaviors, the meta-analysis found inconclusive associations with specific aspects like glycemic control.

Furthermore, beyond the FFM, our analysis identified that individuals with Type D personality tend to exhibit poor adherence across various self-care dimensions, reflecting challenges in diabetes self-management. However, associations between type D personality and glycemic control did not show conclusive evidence. Unexpectedly, findings showcased intriguing associations where high self-esteem and angry hostility displayed similar positive correlations with better glycemic control, while altruism was associated with poorer glycemic control. Such discoveries prompt further exploration into the interplay between these traits and self-care practices among individuals with T2DM.

Our study underscores the pivotal role of personality traits in shaping health-related behaviors and influencing an individual's ability to manage their well-being through lifestyle choices and self-care practices. Personality traits may play an important role in maintaining a healthy lifestyle and stable health in patients with T2DM, directly impacting their discipline to follow a self-care routine based on their particular needs and on feeling alertness to any changes in everyday life. Accordingly, an approach that is guided by the unique needs and personal characteristics of each person with T2DM is of paramount importance. Our findings suggest that healthcare professionals could consider integrating personality trait assessments as a crucial element in enhancing self-care adherence. Assessing personality traits could assist in identifying personalized variations in managing diabetes and in pinpointing individuals who might gain more from specific self-care interventions. However, given the insignificant results of the aggregate estimates and the lack of convincing evidence, there is a need for further, high-quality primary and secondary research to examine these associations in more detail.

## Figures and Tables

**Figure 7:**
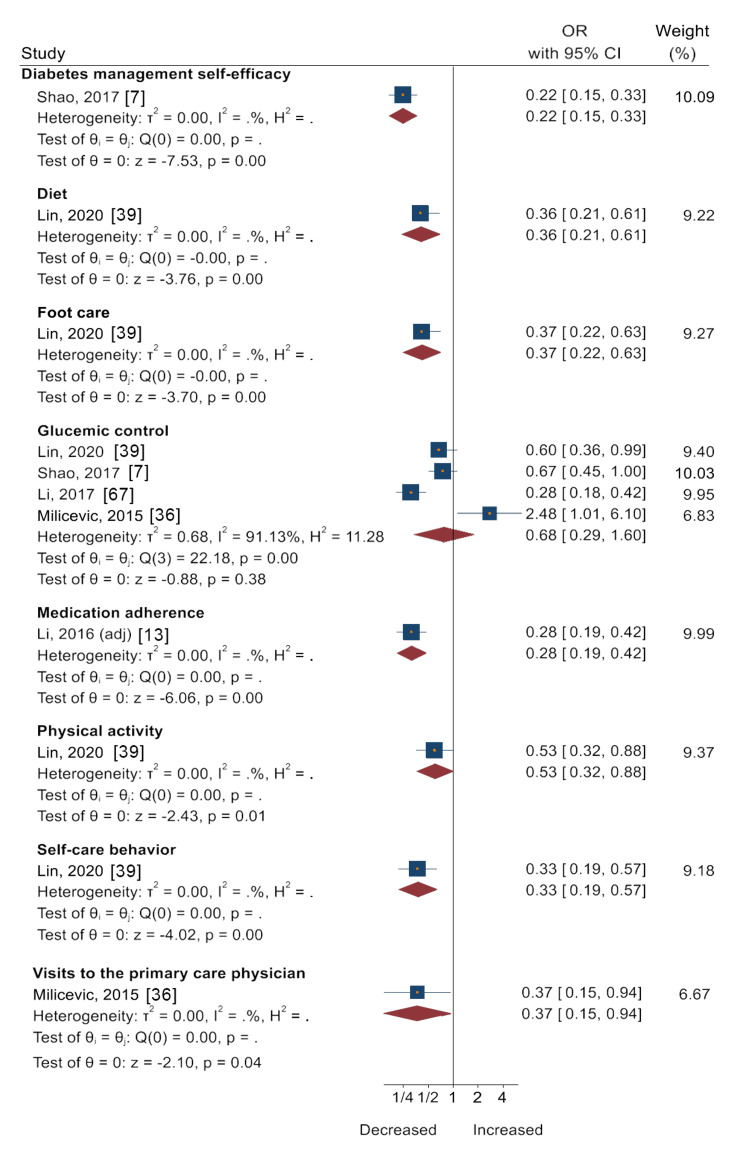
Type D personality and self-care activities in people with type 2 diabetes mellitus Shao et al., 2017 [7]; Lin et al., 2020 [39]; Li et al., 2017 [67]; Li et al., 2016 [13]; Milicevic et al., 2015 [36]

**Figure 8:**
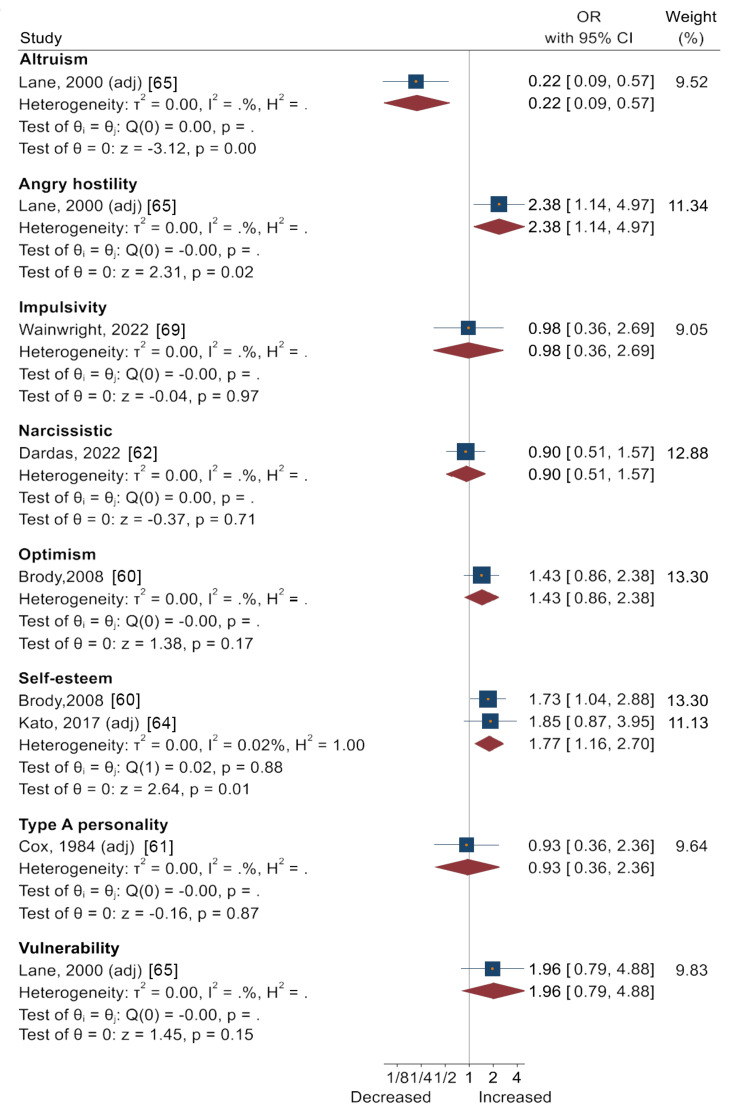
Other personality traits and glycemic control in people with type 2 diabetes mellitus Lane et al., 2000 [65]; Wainwright et al., 2022 [69]; Dadras et al., 2022 [62]; Brody et al., 2008 [60]; Kato et al., 2017 [64], Cox et al., 1984 [61]

**Figure 9:**
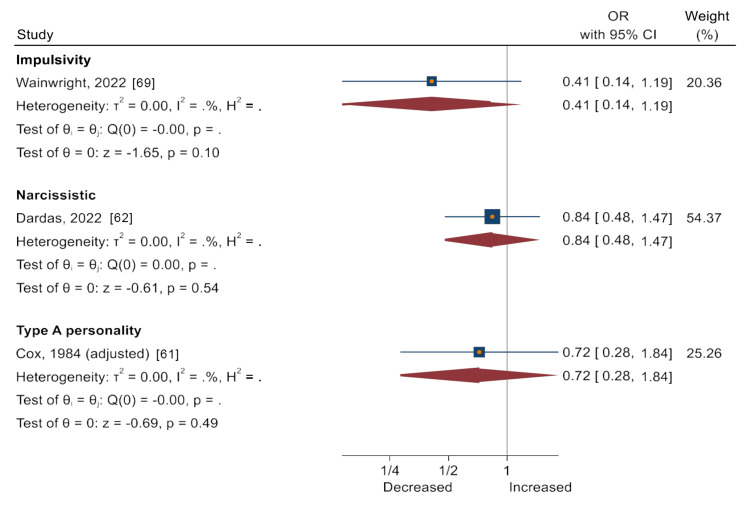
Other personality traits and medication adherence in people with type 2 diabetes mellitus Wainwright et al., 2022 [69]; Dadras et al., 2022 [62]; Cox et al., 1984 [61]

**Figure 10:**
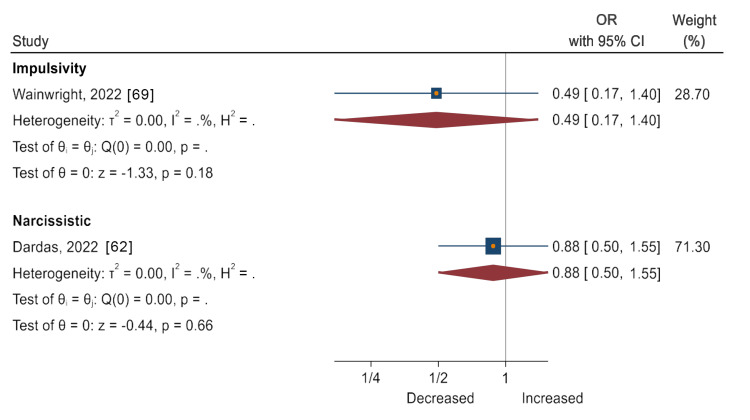
Other personality traits and physical activity in people with type 2 diabetes mellitus Wainwright et al., 2022 [69]; Dadras et al., 2022 [62]

**Figure 11:**
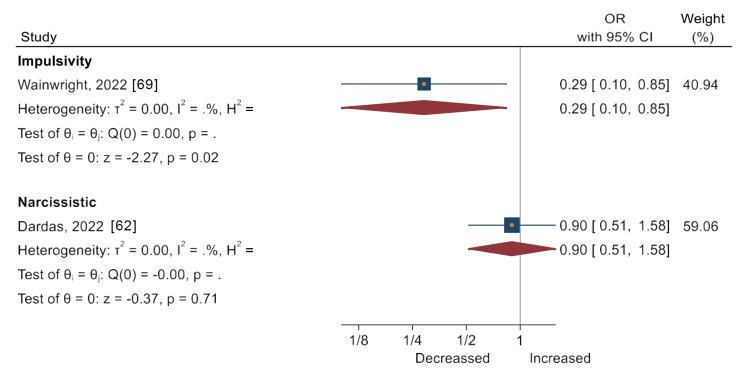
Other personality traits and diet in people with type 2 diabetes mellitus Wainwright et al., 2022 [69]; Dadras et al., 2022 [62]

**Figure 12:**
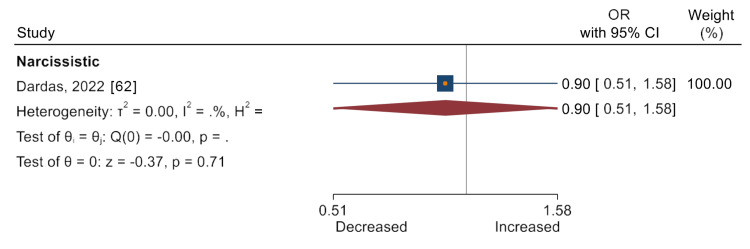
Other personality traits and foot care in people with type 2 diabetes mellitus Dadras et al., 2022 [62]

**Figure 13:**
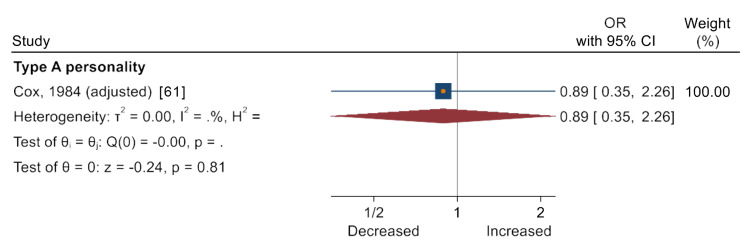
Other personality traits and self-care behavior in people with type 2 diabetes mellitus Cox et al., 1984 [61]

## Appendices

**Table 1:** Search query for all databases

For PubMed
("psychol assess"[Journal] OR ("psychological"[All Fields] AND "assessment"[All Fields]) OR "psychological assessment"[All Fields] OR (("psychologic"[All Fields] OR "psychological"[All Fields] OR "psychologically"[All Fields] OR "psychologization"[All Fields] OR "psychologized"[All Fields] OR "psychologizing"[All Fields]) AND ("profile"[All Fields] OR "profiled"[All Fields] OR "profiler"[All Fields] OR "profilers"[All Fields] OR "profiles"[All Fields] OR "profiling"[All Fields] OR "profilings"[All Fields])) OR ("person s"[All Fields] OR "personable"[All Fields] OR "personableness"[All Fields] OR "personal"[All Fields] OR "personalisation"[All Fields] OR "personalise"[All Fields] OR "personalised"[All Fields] OR "personalising"[All Fields] OR "personality"[MeSH Terms] OR "personality"[All Fields] OR "personalities"[All Fields] OR "personality s"[All Fields] OR "personalization"[All Fields] OR "personalize"[All Fields] OR "personalized"[All Fields] OR "personalizes"[All Fields] OR "personalizing"[All Fields] OR "personally"[All Fields] OR "personals"[All Fields] OR "persons"[MeSH Terms] OR "persons"[All Fields] OR "person"[All Fields]) OR (("person s"[All Fields] OR "personable"[All Fields] OR "personableness"[All Fields] OR "personal"[All Fields] OR "personalisation"[All Fields] OR "personalise"[All Fields] OR "personalised"[All Fields] OR "personalising"[All Fields] OR "personality"[MeSH Terms] OR "personality"[All Fields] OR "personalities"[All Fields] OR "personality s"[All Fields] OR "personalization"[All Fields] OR "personalize"[All Fields] OR "personalized"[All Fields] OR "personalizes"[All Fields] OR "personalizing"[All Fields] OR "personally"[All Fields] OR "personals"[All Fields] OR "persons"[MeSH Terms] OR "persons"[All Fields] OR "person"[All Fields]) AND ("characteristic"[All Fields] OR "characteristics"[All Fields])) OR (("psychologic"[All Fields] OR "psychological"[All Fields] OR "psychologically"[All Fields] OR "psychologization"[All Fields] OR "psychologized"[All Fields] OR "psychologizing"[All Fields]) AND ("characteristic"[All Fields] OR "characteristics"[All Fields]))) AND ("diabetes mellitus, type 2"[MeSH Terms] OR "type 2 diabetes mellitus"[All Fields] OR "type 2 diabetes"[All Fields] OR ("diabetes mellitus, type 2"[MeSH Terms] OR "type 2 diabetes mellitus"[All Fields] OR ("diabetes"[All Fields] AND "mellitus"[All Fields] AND "type"[All Fields] AND "ii"[All Fields]) OR "diabetes mellitus type ii"[All Fields]) OR "t2dm"[All Fields])
For PsycINFO-CINAHL – MEDLINE via EBSCOhost
STX (psychological assessment OR psychological profile OR personality OR personality characteristics OR psychological characteristics) AND TX (type 2 diabetes or diabetes mellitus type II or t2dm)

**Table 2:** Characteristics of included studies Notes: y=Year NR=Not reported, T2DM= Type 2 diabetes mellitus, A1C= Glycosylated haemoglobin, ICD-10= The International Classification of Disease 10th revision, BFI= Big Five Inventory, JAS Type A scale= Jenkins Activity Survey, IPIP= International Personality Item Pool, LSI= Godin leisure-time exercise questionnaire was calculated using the leisure score index, MCMI-III= Millon Clinical Multiaxial Inventory III, SDSCA= Summary of Diabetes Self-Care Activities, NEO-PI= Neuroticism-Extraversion-Openness Personality Inventory, BFI-44= Big five Inventory-44 items, NEO-PI-R= Neuroticism- Extraversion-Openness Personality Inventory-Revised, RCT= Randomized Control Trial, MMAS-8= Moriski Medication Adherence Scale-8, QB4= Quick Big 4, DS-14= Type D scale-14 items, C-DSC= Diabetes Self-Care Scale, BIS-11= The Barratt Impulsiveness Scale-11 items, TIPI-J= Ten-Item Personality Inventory Japanese version

Author, Year	Country	Study design	Sample character ristics	Medication	Mean age	Duration of diabetes	Diabetes diagnostic criteria	%Woman	Exposure	Measure ment of personality	Outcome	Measurement of outcome	Main Finding	Quality
(Abdullah et al., 2020) [37]	Malaysia	Cross- sectional	300	All patients were on oral hypoglycemic medications, 138 of the patients were on insulin therapy, 114 without insulin	63	14 y	People with a diagnosis of T2DM	89	Big-Five personalit y dimension s	BFI	Glycemic control	A1C of ≥7.0%	No psychological factors were associated with poor glycemic control	Good
(Brody et al., 2008) [60]	Georgia	Cross- sectional	200	NR	52.5	1-10 y	People with known a diagnosis of T2DM	70	Self- esteem, Optimism	Rosenber Self- Esteem Scale, 8- item Life Orientation Test	Glycemic control	A1C	Low self- esteem and low optimism have positive relationship with Glycemic control	Good
(Cox et al., 1984) [61]	United States	Cross- sectional	60	All participants were taking insulin	41.7	13.83 y	People with a diagnosis of T2DM	65	Type A behavior	JAS Type A Scale	Glycemic control, Diet, Exercise	A1C	Type A behavior not correlated with A1C	Good
(Davies et al., 2010) [30]	Australia	Prospecti ve study design	74	NR	61	NR	People with a diagnosis of T2DM	57	Big-Five personalit y dimension s	IPIP	Physical activity	LSI	Conscientious ness have positive relationship with physical activity	Good
(Dadras et al., 2022) [62]	Iran	Descripti ve- Correlati onal	160	NR	69.4	NR	Based on the laboratory tests and medical records	38.1	Depressed, Narcissisti c	MCMI- III	Diet, Physical activity, Medication adherence, Glycemic control, Foot care	The Summary of Diabetes Self-Care Activities measure (SDSCA)	There is a negative relationship between personality profile and self-care	High
(Esmaeilinasab et al., 2016) [15]	Tehran	Cross- sectional	400	287 used pills and 113 had insulin injections	51	10.1 y	People with a diagnosis of T2DM	36	Big-Five personalit y dimensions	NEO-PI	Glycemic control	A1C	Extraversion, Conscientious ness, Neuroticism associated with A1C	High
(Fisher et al., 2014) [31]	United States	RCT	392	70 were taking insulin	56.11	> 1 y	People with a diagnosis of T2DM	53.8	Conscienti ousness	Conscien tiousness was assessed using a nine-item scale	Medication adherence, Physical activity	Eight-item Hill–Bone Compliance Scale	Conscientious ness was a significant predictor of change in medication adherence	Some concerns
(Hazrati-Meimaneh et al., 2020) [63]	Iran	Cross- sectional	495	348 were not injection users, 147 were injection users	57.59	ΝR	People with a diagnosis of T2DM	73.5	Big-Five personalit y dimension s	NEO-PI- R	Medication adherence, Self-care behavior	SDSCA, MMAS-8	Neuroticism, extraversion, and agreeableness significantly contributed to Medication Adherence, but not to Self-Care Behavior	Good
(Kato et al., 2017) [64]	Japan	Cross- sectional	209	73 were injection therapy	60.2	13.2y	People with a diagnosis of T2DM	19.6	Self- esteem	Self-Esteem Scale by Rosenberg	Glycemic control	A1C	Low self-esteem associated with poorer self-care	Good
(Lane et al., 2000) [65]	United States	Longitud inal cohort	105	Most participants (73%) were receiving medication for the treatment of their diabetes	55.9	5.0y	People with a diagnosis of T2DM	44	Big-Five personalit y dimension s	NEO-PI- R	Glycemic control	A1C	Lower average blood glucose values at baseline were associated with higher scores for the personality domain of neuroticism and several specific traits including anxiety, angry hostility, depression, self- consciousness, and vulnerability but were associated with lower scores for the trait of altruism. Results were similar for A1C but were not as strong	High
(Lee and Li, 2021) [66]	Taiwan	Cross- sectional	214	NR	55.64	8.86y	People with a diagnosis of T2DM	57	Big-Five personalit y dimension s	QB4	Glycemic control	A1C of ≥7.0%	Neuroticism associated with higher A1C level (3.199 times more than those with the “openness- extraversion” personality trait)	Good
(Li et al., 2017) [67]	China	Longitudinal cohort	330	NR	57.23	7.87y	ICD-10	52.1	Type D personality	DS14	Medication adherence	A1C	Type D personality associated with higher A1C value	Good
(Li et al., 2016) [13]	China	Longitud inal cohort	330	NR	59.77	7.87y	ICD-10	50.2	Type D personalit y	DS14	Medication adherence	MMAS-8-CN, blood glucose	Type D personality predicts poor medication adherence before and after controlling for covariates whe n it was analyzed as a categorical variable. Type D personality was not associated with medication adherence when analyzed as a continuous variable	Good
(Lin et al., 2020) [39]	Taiwan	Cross- sectional	198	55 taking an oral antidiabetic drugs, 10 only insulin, 114 oral antidiabetic, and insulin, 1 no treatment, 18 only diet and exercise	51.2	Under 6 months- Above10 years	American Diabetes Associatio n (2016)	37.4	Type D personalit y	DS14- TR	Self-Care Behaviors (dietary, physical activity, Glycemic control, and foot care)	C-DSC	Type D personality had negative correlation with self-care behaviors (including dietary, physical activity, higher or lower blood glucose prevention and treatment, medical and blood glucose monitor, and foot-care)	Good
(Mendoza-Catalán et al., 2022) [12]	Mexico	Cross- sectional	197	NR	53.1	9.0 y	People with a diagnosis of T2DM	74.6	Big-Five personalit y dimension s	BFI	Self-care activities	SDSCA	People with greater openness and conscientiousn ess were associated with greater compliance with self-care. Conversely, neuroticism was inversely associated with self-care in patients with T2DM	Good
(Milicevic et al., 2015) [36]	Bosnia and Herzego vina	Cross- sectional	91	NR	62.41	NR	ICD-10	56	Type D personalit y	DS-14	Complianc e	Frequency of blood glucose assessment and frequency of encounters with the primary care physician	Patients with type D personality were less compliant in terms of the visits to the primary care physician, although they were more prevalent among those who were compliant regarding the frequency of blood glucose assessment	Good
(Novak et al., 2017) [68]	United States	Cross- sectional	117	NR	57.4	NR	People with a diagnosis of T2DM	42.7	Neuroticis m	BFI	Diet, Physical activity	SDSCA	Neuroticism associated with dietary and exercise adherence	High
(Phillips and Guarnacci, 2016) [29]	United States	Cohort	2301	NR	69.49	NR	People with a diagnosis of T2DM	54.4	Big-Five personalit y dimensions	Five factor model	Glycemic control	A1C	Personality traits associated with diabetic control	Good
(Shao and Yin, 2017) [7]	China	Cross- sectional	97	Participants taking diabetic treatment	61.77	NR	People with a diagnosis of T2DM	57.9	Type D personalit y	DS14	Glycemic control and self - efficacy	A1C and self- efficacy scale designed by Lorig et al	Type D Pearsonality associated with poor glycemic control and less self-efficacy	Good
(Skinner et al., 2014) [11]	Australia	Longitud inal cohort	1551	1457 taking insulin and oral medication	65.8	9.0	People with a diagnosis of T2DM without insulin	47.1	Big-Five personalit y dimension s	BFI44	Glycemic control	A1C	No independent association between personality traits and A1C	Good
(Williams et al. 2002) [40]	United States	Within subjects repated measure ment	94	69 were taking oral medication but none with insulin	56.9	5.0	People with a diagnosis of T2DM	43.6	Neuroticis m	Neurotici sm scale of the NEO-PI-R	Glycemic control	Lifescan One- Touch II glucometer	Neuroticism related to overestimation of blood glucose	High
(Wainwright et al., 2022) [69]	United States	Cross- sectional	50	NR	51.03	NR	Self- reported diagnosis	71.2	Impulsivit y	BIS-11	Diet, Physical activity,Medication adherence, Glycemic control	SDSCA	Impulsiveness only negatively predicted exercise and diet adherence	Good
(Yasui-Furukori et al., 2020) [89]	Japan	Cross- sectional	504	448 were taking an oral hypoglycemic agent and 237 were receiving insulin therapy.	63.9	NR	Received treatment for at least 1 year	41.9	Big-Five personalit y dimension s	TIPI-J	Glycemic control	A1C	None of the personality factors, including neuroticism, were found to be associated with A1C after adjustment for confounders	High
